# Bridging the gap to advance the care of individuals with cancer: collaboration and partnership in the Cardiology Oncology Innovation Network (COIN)

**DOI:** 10.1186/s40959-022-00129-y

**Published:** 2022-02-09

**Authors:** Sherry-Ann Brown, Craig Beavers, Hugo R. Martinez, Catherine H. Marshall, Iredia M. Olaye, Avirup Guha, David Cho, Alison Bailey, Carmen Bergom, Neha Bansal, Brenton Bauer, Richard K. Cheng

**Affiliations:** 1 Cardio-Oncology Program, Division of Cardiovascular Medicine, Medical College of Wisconsin, 8701 Watertown Plank Road, Milwaukee, WI 53226 USA; 2 University of Kentucky College of Pharmacy, Lexington, KY USA; 3 The Heart Institute at Le Bonheur Children’s Hospital, University of Tennessee Health and Science Center, Memphis, TN USA; 4 St. Jude Children’s Research Hospital, Memphis, TN USA; 5 Sidney Kimmel Comprehensive Cancer Center, Johns Hopkins University, Baltimore, MD USA; 6 Johns Hopkins Ciccarone Center for the Prevention of Cardiovascular Disease, Division of Cardiology, Johns Hopkins University School of Medicine, Baltimore, MD USA; 7 Division of Clinical Epidemiology, Department of Medicine, Weill Cornell Medicine, New York, NY USA; 8 Cardio-Oncology Program, Division of Cardiology, Department of Internal Medicine, Medical College of Georgia at Augusta University, Augusta, GA USA; 9 University of California, Los Angeles, Division of Cardiovascular Medicine, Los Angeles, CA USA; 10 Center for Heart, Lung, and Vascular Health at Parkridge, HCA Healthcare, Chattanooga, TN USA; 11 Department of Radiation Oncology, Washington University in St. Louis, St. Louis, MO USA; 12 Cardio-Oncology Center of Excellence, Washington University in St. Louis, St. Louis, MO USA; 13 Division of Pediatric Cardiology, Children’s Hospital at Montefiore, Albert Einstein College of Medicine, Bronx, NY USA; 14 COR Healthcare Associates, Torrance Memorial Medical Center, Torrance, CA USA; 15 Cardio-oncology Program, Division of Cardiology, University of Washington, Seattle, WA USA

## Abstract

Cardiovascular diseases and cancer continue to be the two leading causes of death in the United States. While innovations in artificial intelligence, digital health, and telemedicine may revolutionize cardio-oncology clinical practice, barriers to widespread adoption continue to exist. The most effective way to advance these technologies is through a broad range of stakeholders sharing a common vision. Additionally, as we enter the digital era in healthcare, we must help lead this charge for the benefit of our cardiology and oncology patients. Bolstering collaborations in cardiology and oncology is key, in partnership with technology firms, industry, academia, and private practice, with an emphasis on various forms of innovation. The ultimate goal is to connect our patients and their health to informatics-based opportunities to advance cardiovascular disease prevention in cancer patients. We have established the Cardiology Oncology Innovation Network in accordance with this vision, to develop new care delivery options through the use of innovative technological strategies. Our tripartite mission – innovation, collaboration, and education – aims to increase access to and expertise in digital transformation to prevent cardiovascular diseases in cancer patients. Here we describe network initiatives, early accomplishments, and future milestones.

## Introduction

Cardiovascular disease (CVD) and cancer remain the two leading causes of death in the United States, despite improved prevention and treatment strategies for both conditions [1]. For some cancer survivors, the risk of cardiovascular death surpasses the risk of death due to cancer, making preventive cardio-oncology critical for long-term health and well-being [2, 3]. While there has been increasing focus on the cardiovascular effects of cancer and cancer treatment, less is known about effective strategies and tools for preventing CVD among these patients [4]. Digital health technologies and innovations such as artificial intelligence have the potential to transform cardio-oncology clinical practice, but barriers to widespread use persist [5]. The best method to support desired healthcare advances in digital medicine (e.g., cardio-oncology) is through a shared vision among a broad range of stakeholders [6]. Further, as we enter into the age of digital healthcare transformation, for the sake of our patients in Cardiology and Oncology, we need to help lead this charge. We must strengthen our collaboration in Cardiology and Oncology – two specialties that are “stronger together” at the intersection affecting our patients in cardio-oncology [7]. We must build bridges among technology companies, industry, academia, and private practice, with an intense focus on innovation (i.e., digital health, mobile health, telemedicine, telehealth, informatics, precision health, computational medicine, artificial intelligence, machine learning, deep learning, natural language processing, neural networks, and social media, among others). The ultimate goal is connecting our patients and their health with informatics-based opportunities to advance the prevention of CVD in patients with cancer.

## Cardiology Oncology Innovation Network (COIN)

**Fig. 1:**
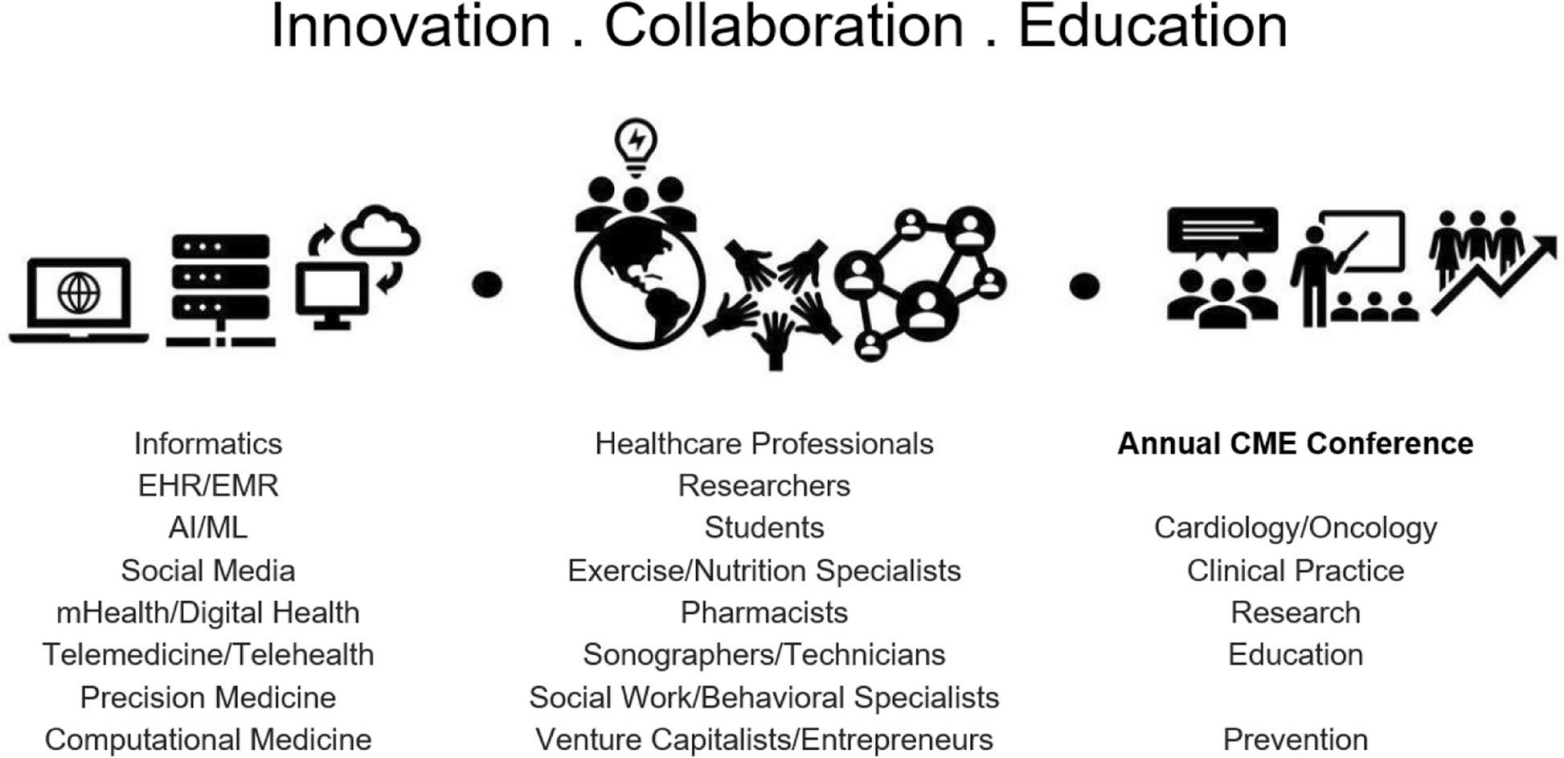
Cardiology Oncology Innovation Network Tripartite Mission. AI = artificial intelligence; CME = continuing medical education; EHR = electronic health records; EMR = electronic medical record; mHealth = mobile health; ML = machine learning

The rapid expansion of cardio-oncology over recent years continues to challenge clinicians to digest, interpret, and share an increasing amount of information efficiently. The need for health informatics paired with innovative means of capturing and utilizing patient-centric data in cardio-oncology has never been greater. Observational studies have reported improved quality of healthcare indicators after implementation of practice guidelines established and disseminated by clinical networks [8]. With this tripartite mission – innovation, collaboration, and education – our goal is to increase access and expertise on digital transformation to prevent CVD in patients with cancer (Fig. 1). Multidisciplinary contribution to the generation of research initiatives is of utmost importance for this network to continue innovating the field of cardio-oncology and making current and future innovations available to our patients.

## Innovation

In line with this vision, we have established the Cardiology Oncology Innovation Network (COIN). COIN aims to use innovative technological strategies to create and improve care delivery options, knowledge, and access to preventive and diagnostic cardio-oncology for the growing population of cancer survivors. These innovative methods will help us develop optimal protocols and techniques with research to prevent, monitor, and mitigate acute and chronic cardiovascular effects from cancer therapies, during and shortly after treatment (particularly with many of the new drugs), as well as throughout the duration of survivorship long after cancer therapy is completed. Innovative tools and research for early and long-term surveillance in the years after treatment will be pursued to provide comprehensive healthcare before, during, and after a cancer diagnosis.

## Collaboration

COIN is committed to setting the foundation to prevent and manage CVD by forming relationships with other medical disciplines, especially working in collaboration with oncologists to redesign treatments to minimize cardiac risk without compromising cancer outcomes. We aim to increase collaborative efforts among thought leaders worldwide in the field, including health care professionals, researchers, students, industry members, and entrepreneurs. Promoting these efforts will improve patient outcomes as healthcare moves forward into an increasingly technological-centered future.

## Education

Through the network, education of our healthcare professionals and patients regarding cardio-oncology is vital to alter the trajectory of CVD in individuals with a current or prior history of cancer. We intend to generate new knowledge that will be disseminated through annual continuing medical education (CME) conferences and utilize asynchronous platforms for ongoing learning.

COIN offers grassroots opportunities to have a wide diversity of patients representing myriad cultures, demographics, and geography, including rural and urban populations, to address healthcare disparities and inequities. COIN leverages health informatics to harness the power of data for medical providers making strategic healthcare decisions. The platform will relay information in an accessible manner to all centers and sites. Innovative education pathways and platforms are promoted by creating and sharing didactic material, emphasizing patient care delivery with centralized resources, and adopting a core innovation curriculum. Sustainability will be based on funding from foundations and investigator-initiated projects promoting technology targeted to various efforts in improving cardio-oncology care.

We will develop data-backed tools and applications that practicing clinicians on the front lines of care will be able to utilize, including decision aids that will streamline the application of scientific statements and clinical guidelines within cardio-oncology. The network provides thought leadership, content expertise for high-impact topics, and programming accessible at point-of-care, and engagement to contribute actionable insights that can be readily translated to clinical practice.

Network goals are to:

- Initiate a multi-regional, multi-institutional network to leverage digital and technological innovation built upon collaboration;
- Implement education of healthcare workers and patients, and improve the cardiac outcomes of cancer survivors;
- Develop patient-centered teamwork of diverse professionals in cardiology and oncology;
- Provide new avenues for collaboration on innovative projects in cardio-oncology;
- Offer professional development tools for education on innovation in cardio-oncology;
- Establish organizational sustainability with a pipeline of innovators and researchers, including medical and graduate students, as well as clinical and research fellows actively involved in the network and summit;
- Advance innovation and knowledge through research to optimize cardiovascular care and outcomes and transform the cardiovascular health of cancer patients and survivors;
- Research needs of professionals and patients in cardiology and oncology and assess effectiveness of novel interventions developed through the network;
- Foster research collaborations to advance innovation in cardio-oncology;
- Shape care delivery and disseminate actionable knowledge.

These goals will be re-evaluated iteratively to reflect growth and integrate progression within our network.

## Milestones

**Fig. 2:**
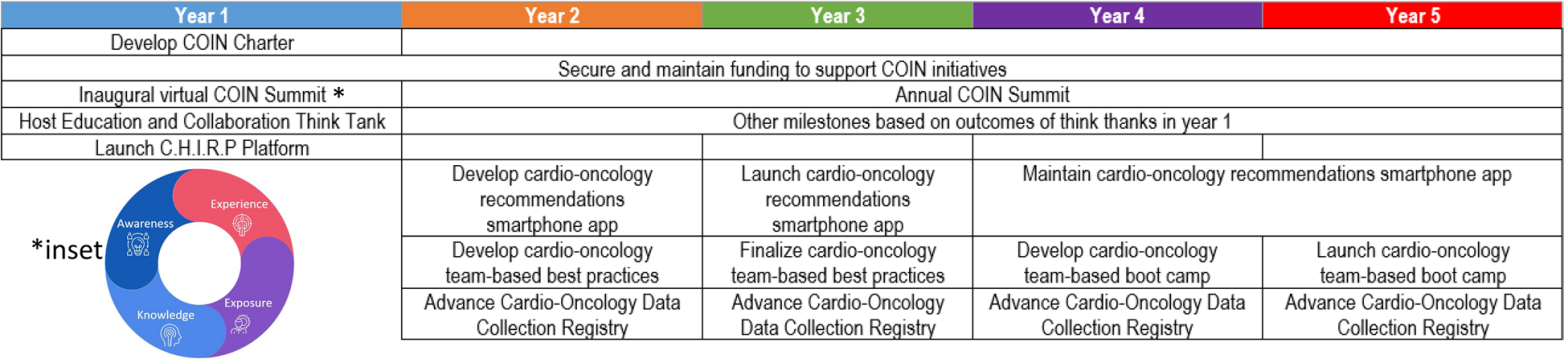
Cardiology Oncology Innovation Network 5-year Strategic Plan. Inset, Guiding Principles of the Cardiology Oncology Innovation Network

COIN membership has established incremental milestones to fulfill its mission and provide value to the cardio-oncology community. These initially proposed milestones encompass the spirit of collaboration, innovation, and education which serve as the foundation for COIN. Furthermore, COIN will expand or modify these milestones iteratively based on community needs and scientific advancement. We have already made substantial progress with achieving prespecified milestones in the first year (Fig. 2), including the successful launch of the virtual inaugural COIN Summit to disseminate awareness, knowledge, exposure, and experience with innovation and technologies in cardio-oncology (Fig. 2 inset).

## Sustainability pipeline

We have established organizational sustainability with a pipeline of innovators and stakeholders. The pipeline captures a wide spectrum of individuals, from medical, undergraduate, and graduate students, medical residents, clinical and research fellows, to established scientists and clinicians, as well as other health care workers, and technology and industry experts. Individuals from throughout the pipeline serve on the steering committee. We also involve the spectrum of members in innovative educational activities in the network, including topics covered through the network’s online CME/Bootcamp currently being developed. A range of innovation topics are represented, encompassing artificial intelligence, digital health, mobile health applications, telemedicine/telehealth, and precision health/medicine. To help shape care delivery and disseminate actionable knowledge, we engage both technology or industry companies and academic and private health professional institutions as active participants in the annual CME COIN Summit. Further, partnerships optimizing pediatric cardio-oncology care and research have been forged. Emphasis has been placed on preventive initiatives at all ages and stages of cardiovascular management. Advancing team-based program development and pathways/protocols are critical in our network, demonstrating care team roles with individuals utilizing the full extent of their training.

Our current membership consists of individuals associated with 60 academic hospitals/health systems, 5 private/community practices, and 10 industry company partners. Network members in cardiology, oncology, and particularly cardio-oncology, as well as industry partners in innovation, technology, and regulatory affairs, along with trainees at various stages, have been invited by email or social media. All interested parties are broadly encouraged to join us in these endeavors; membership is continuous and open to all. Professionals or trainees from around the world can directly join the network via our website or social media accounts (CardioOncCOIN.Org, CardioOncCOIN@Gmail.Com, @CardioOncCOIN on Twitter, LinkedIn).

## Early outcomes

From among the diverse membership, 10 of our most actively engaged academicians estimated their current pre-existing institutional groups’ collaborative achievements relevant to the tripartite COIN mission (innovation, collaboration, education) on related topics such as applying innovation (digital health, mobile health, telemedicine, telehealth, informatics, precision health, computational medicine, artificial intelligence, social media, and so on) to cardiology and oncology clinical practice, research, or education over the past 6 months prior to submission of this publication. The results of this sample data are as follows. Overall, the research groups of these 10 COIN academicians combined currently have 514 papers published or currently accepted/in press, 41 papers currently in review/revision, 88 internal review board (IRB) protocols submitted or approved, 56 external grants obtained, 11 internal grants obtained, 32 external grant applications submitted, and 13 internal grant applications submitted. This robust baseline productivity among our members provides a solid foundation for synergy in future network collaborative research efforts. Our network members have already begun to produce research and scholarly output together. A cardio-oncology American Heart Association Scientific Statement is currently in press that has co-authors and reviewers that were independently extracted from across COIN membership. Multiple IRB protocols are in process for academia-industry prospective and retrospective research projects.

With this backdrop, we have already taken several concrete steps towards achieving the goals of our tripartite mission. We have begun launching the Connected Healthcare Innovation Research Program (C.H.I.R.P) platform. C.H.I.R.P. connects our patients and clinicians in cardio-oncology with innovative mobile health applications and digital health devices and wearables utilizing artificial intelligence to optimize cardiovascular health and wellness. Approximately 10 industry partners are participating in retrospective and prospective collaborative research studies, as well as providing innovative tools and products for clinical care to enhance the fitness, wellbeing, and hospital environment of patients. In addition to C.H.I.R.P., we have partnered to help initiate development of the multi-center informatics-driven global cardio-oncology registry optimized for Precision Health, in collaboration with the American College of Cardiology and International Cardio-Oncology Society cardio-oncology international advocacy and collaborative network.

The global cardio-oncology registry will foster collaborative database research to better understand risk factors for cardiovascular conditions resulting from cancer therapies in various geographic regions and demographics. In the first few months, data will be collected from up to 25 US locations to optimize workflow. The registry will grow over time, with the initial pilot phase focusing on individuals diagnosed with breast cancer. Subsequently, hematologic cancers will then be included, followed by other cancers. Data collection will include ECG, cardiac imaging (such as echocardiography), biomarkers (such as troponin and NT-proBNP), details on cancer type and staging, cancer therapy, cardiovascular morbidity, mortality, and sociodemographic parameters. All data will be stored in a global REDCap Cloud data repository hosted by Cleveland Clinic. Data will be entered securely into REDCap Cloud by clinicians, trainees, and supporting staff at more than 100 participating academic and community sites. The registry IRB protocol includes standard institutional requirements for data safety. All sites will process their IRB approval and complete their data sharing agreement prior to inclusion in the database. Data will be maintained in REDCap Cloud, a cloud-based electronic data capture software that stores de-identified data on an online, secure, encrypted and password-protected server [9]; REDCap software is validated and abides by FDA and HIPAA regulations. The registry is designed to be longitudinal, in order to collect data over several years to capture late effects on the cardiovascular system from cancer therapy. Precision medicine, artificial intelligence, and other innovative informatics tools will be incorporated into the registry to help assess and address prevention, management, survivorship, and socioeconomic and racial disparities in cardio-oncology. Integrated and automated data extraction on parsimonious data points from the electronic healthcare system will help increase sustainability. Such a prospective, shared registry with harmonized case ascertainment is critical for assuring high-quality data across sites for research collaboration and discoveries. This registry will leverage the existing COIN and national society infrastructure to ensure efficient operationalization with incorporation of informatics and precision medicine.

Notably, we have also hosted the inaugural annual COIN ThinkTank and the inaugural annual COIN Summit. The inaugural COIN Summit was held in December 2020, with almost 100 international leaders in cardiology, oncology, and industry in attendance. The Summit featured clinicians and scientists, many of whom hold relevant leadership positions in the American College of Cardiology, American Heart Association, American Society for Preventive Cardiology, American Society for Clinical Oncology, American Society for Radiation Oncology, International Cardio-Oncology Society, World Health Organization, and United Nations, and also a previous winner of Shark Tank. Continuing professional development topics addressed with learning objectives at the Summit included informatics, artificial intelligence, big data, machine learning, and natural language processing. Subsequent to this, the inaugural COIN ThinkTank was held in August 2021, with 30 key stakeholders as cardiology and oncology clinical and research experts and trainees, as well as industry partners, and representing leading cardio-oncology programs in 5 countries and 15 US states.

## Future engagement goals

Over time, we will continue to catalog network members’ collaborative accomplishments highlighting deliverables, metrics, and outcomes to advance innovation for our patients in cardio-oncology. We will continue to increase the number of academic hospitals/health systems, private and community practices, and industry company partners, with participants encompassing a comprehensive range of professions and career stages. We will continuously build and sustain our innovator pipeline, with diverse expertise in informatics, artificial intelligence, digital health, mobile health, telemedicine/telehealth, precision health/medicine, computational medicine, and social media.

We will utilize our partnerships with professional societies to share insights and outcomes at scientific sessions, providing further opportunities to grow COIN membership and output.

**Fig. 3:**
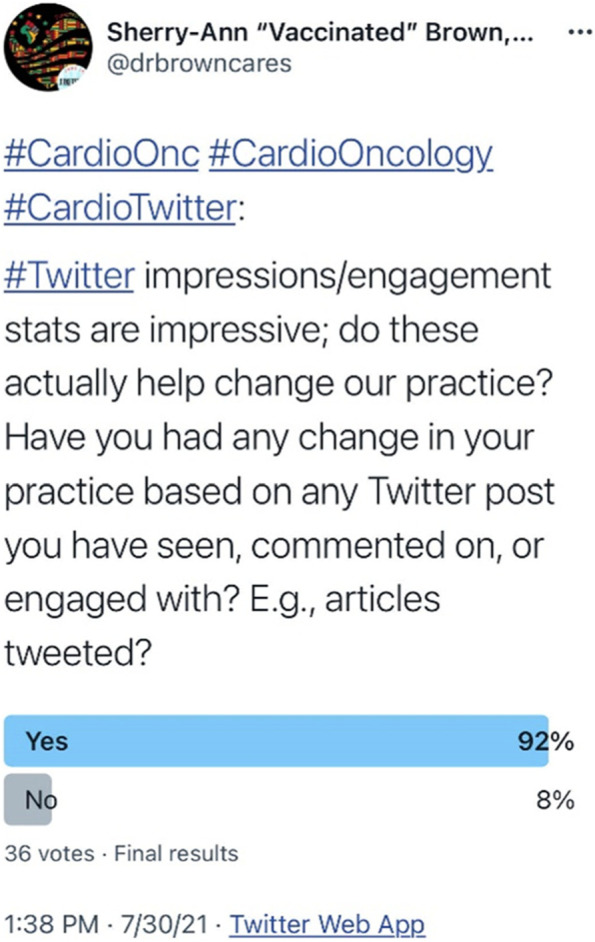
Cardiology Oncology Social Media Potential Benefit

The cardio-oncology community indicated in a recent poll that social media posts, particularly those describing new scientific or medical journal articles, can be helpful for informing patient care (Fig. 3). More than 90% of Twitter users responding to the poll reported that such social media posts have led to changes in their clinical practice. This suggests that social media can be leveraged for education and subsequent patient care. In our network, we will continue to develop new studies and insights regarding social media to optimize these platforms for impacting patient care, research, innovation, collaboration, and education of both health care professionals and patients.

We will monitor engagement statistics for mobile health applications (downloads, time spent on mobile health application, user reviews) developed by the network, as well as those produced by our C.H.I.R.P. industry partners, noting associations with short- or long-term clinical outcomes. As with any initiative in technology, standardization across different platforms may bring challenges. This may take the form of different electronic health records across numerous institutions, or various mobile health applications used by our patients (e.g., a range of blood pressure or other digital health software tools). Establishing COIN as a central initiative may help standardize these various technologies and guide our patients as they navigate the complex digital and increasingly technological healthcare landscape.

## Conclusion

We will continue to build a network that utilizes innovation and knowledge to optimize cardiovascular care and outcomes, shapes the cardiovascular health of cancer patients and survivors, and assesses the impact of innovation in cardio-oncology. In the network, we will seek to increase the number of hospitals and practices implementing innovations related to cardio-oncology, and foster the uptake of innovations by cancer patients and survivors. Importantly, we will maintain a culture of fostering collaboration and excellence among an increasing number of patient-centered and team-based diverse network professionals. Through our shared vision of prevention and innovation, we hope to meaningfully improve the heart health of our cancer patients and survivors who are at risk for cardiovascular adverse events.

## Data Availability

Not applicable.

## Abbreviations

CVD: Cardiovascular Disease
C.H.I.R.P.: Connected Health Innovation Research Program
CME: Continuing Medical Education
COIN: Cardiology Oncology Innovation Network
